# Incidence and impact of stroke during Hajj: Results of 2015 Hajj stroke registry

**DOI:** 10.17712/nsj.2017.3.20160246

**Published:** 2017-07

**Authors:** Mohammed A. Almekhlafi, Maher A. Alhazmi, Sarah S. Alsulami, Soha A. Almorsy

**Affiliations:** *From the Department of Neurology (Almekhlafi), Faculty of Medicine, King Abdulaziz University, Jeddah, from the Research Center (Alhazmi, Almorsy), King Abdullah Medical City, from the Department of Internal Medicine (Alsulami), Alnoor Specialist Hospital, Makkah, Kingdom of Saudi Arabia, and from the Medical Pharmacology Department (Almorsy), Faculty of Medicine, Cairo University, Cairo, Egypt*

## Abstract

**Objective:**

To investigate the incidence of stroke among pilgrims and describe factors associated with presentation and management during Hajj. Physical stress, age and vascular risk factors render pilgrims at a high risk of stroke.

**Methods:**

This is a prospective registry of all acute stroke patients admitted to 6 hospitals during the 2015 Hajj season from 9 September to 4 November. Participating hospitals were King Abdullah Medical City (KAMC), Al-Noor, King Abdulaziz hospital, King Faisal hospital, Hira, and Mena general hospitals; all in Makkah city. Stroke diagnosis was confirmed by imaging. Clinical, demographic and outcome variables were collected.

**Results:**

The incidence of stroke during 2015 Hajj season is 8.9/100,000; 186 cases. The peak incidence was noted on the day after Arafat. Saudis represented 17.2% while 36% were females. Hemorrhagic stroke occurred in 35 patients (18.8%). The mean age was 60.8±12.9 years while the most common risk factors were hypertension (57%) and diabetes (40.9%). Only 5 patients received intravenous tissue plasminogen activator. The median length of hospital stay was 4 days. Twenty-one patients (11.3%) died during hospitalization. The only factor that approached significance in predicting mortality was hemorrhagic stroke (odds ratio of 1.62; 95% CI: 0.97 to 2.70; p=0.063).

**Conclusion:**

Stroke is a major health burden during Hajj. Educational programs for pilgrims and healthcare workers together with protocol-driven care are expected to positively impact stroke care.

Hajj, the pilgrimage, to Makkah in Kingdom of Saudi Arabia is one of the main rituals of Islam. It is considered obligatory for each Muslim at least once in a lifetime. Annually, a huge number of pilgrims, belonging to more than 150 countries, perform Hajj. Delivering healthcare services in Hajj is an enormous challenge as a large number of pilgrims of various ages and health status gather in a relatively small area for a short period of time.

Stroke is a leading cause of death and long-term neurologic impairment and functional disability. Contrary to the developed countries, there is a paucity of research on stroke in Kingdom of Saudi Arabia,^1^ and no study yet has evaluated stroke care during Hajj in a comprehensive fashion. Most of the studies conducted in Hajj have mainly focused on infectious and heat related diseases.^2^ Only one study discussed stroke during Hajj but was restricted to patients from a single nationality.^3^ A clinical registry is a database that collects data on clinically important events related to a particular population or condition, which is analyzed and disseminated in a report. These data allow healthcare organizations to identify problems with healthcare, develop interventions according to the identified problems, and monitor their progress after implementation of a chosen intervention.^4^ In an effort to understand how stroke care during Hajj is delivered, a Hajj stroke registry was developed. This registry data will hopefully improve stroke care in future Hajj seasons. The objective of the study is to report and analyze stroke cases diagnosed during Hajj and to describe important aspects that are known to determine prognosis in such cases. This would help to know whether clinical presentation and/or management of stroke cases that are discovered under Hajj settings differ from those that are diagnosed under standard circumstances.

## Methods

This is cross sectional study design carried out from September 9, 2015 to November 4, 2015, we prospectively collected patient level data admitted to 6 participating hospitals in Makkah. These hospitals were King Abdullah Medical City (KAMC), Al-Noor, King Abdulaziz hospital, King Faisal hospital, Hira, and Mena general hospitals; all in Makkah city, Kingdom of Saudi Arabia. All patients with acute ischemic or non-traumatic hemorrhagic strokes were included. We excluded patients with traumatic brain injuries, and non-pilgrimage patients. We collected data on patients’ characteristics, diagnostics testing, treatments and outcomes. Data collection was carried out by healthcare volunteers and supervised by a stroke neurologist. To obtain high data quality, immediate data entry was obligatory. This study used the “Dendrite” web-based registry system (Dendrite Clinical Systems LTD, 2015). The registry featured online plausibility checks and help. Quality assessments were performed in terms of daily or weekly checks of registry entries. The transmitted data were encrypted, and the Hajj stroke registry collected only the minimum of the demographic data in order to maintain patients’ anonymity.

Data were then imported from the registry system into Statistical Package for the Social Sciences version 23 (IBM Corp, Armonk, NY, USA) and saved in an SPSS system file to which variable labels and value labels were added. Univariable distributions were examined for anomalies, and errors were corrected. Summary statistics were obtained as appropriate. Continuous variables were compared between the study subgroups using student’s T test while categorical variables were compared using Chi Square test. A multivariable logistic regression model was fit to explore the predictors of variable outcome measures. A significance level of <0.05 was used for all tests. Approvals from the Institutional Review Board and Ministry of Health were obtained.

## Results

A total of 186 stroke cases were admitted to the 6 participating hospitals. Based on the official numbers of total pilgrims (2,084,238), the incidence of stroke during 2015 Hajj season is 8.9/100,000. The peak incidence was noted on the day after Arafat.

Saudis represented 17.2% while 36% were females. The top other nationalities affected were Indian (10.2%), Indonesian (5.9%), Egyptians and Pakistanis (4.3% each). The mean age at the time of stroke was 60.8±12.9 years, while the most commonly reported risk factors were hypertension (57%) and diabetes mellitus (40.9%). **Table 1** shows the different characteristics of patients according to nationality (Saudis versus others). There was no significant difference in the proportion of patients with HTN or with DM in the Saudi versus other nationalities. The Body Mass Index (BMI) could be calculated for 109 patients. The mean BMI was 29.0±27.3 and 23.9% met the WHO definition of obesity (BMI ≥ 30). Hemorrhagic stroke occurred in 35 patients (18.8%). These were mostly deep basal ganglionic hemorrhages (51.4%) followed by lobar hemorrhage (31.7%). **Table 2** shows the locations of haemorrhage in cases of hemorrhagic stroke. Almost all cases (98.9%) of stroke occurred outside hospitals, while the place of onset could not be verified in 2 cases. None of the cases had confirmed hospital onset. Patients were brought to the hospital via emergency medical services in 48.5% of the time, and by private transport in 36.4% of the cases. Transfer between hospitals was required in 15.2% of the patients; mostly as a part of the KAMC Hajj Stroke Hotline program.

**Table 1:** The characteristics of registered stroke cases.

Variables	All cases N=186	Saudis n=32	Non-Saudis n=154	*P*-value
*Age*				
Mean ± SD	60.7±12.9	60.8±16.7	60.7±12.1	0.815
Median (IQR)	61.4 (51.9-70.7)	63.3 (48.3-75.3)	60.7 (54.4-69.9)	
*Gender*				
Males n (%)	119 (64)	23 (71.9)	96 (62.3)	0.306
*BMI*	(n=109)	(n=17)	(n=92)	
Mean±SD	29.0±27.3	43.8±68.0	26.3±4.9	0.584
Median (IQR)	25.7 (23.4-29.4)	26.7 (23.4-30.3)	25.6 (23.2-29.4)	
*Risk Factors*				
CAD	23 (12.4)	2 (6.3)	21 (13.6)	0.248
DM	110 (40.9)	15 (46.9)	61 (39.6)	0.447
HTN	106 (57)	18 (56.3)	88 (57.1)	0.926
AFIB	5 (2.7)	1 (3.1)	4 (2.6)	0.867
Hyperlipidemia	4 (2.2)	1 (3.1)	3 (1.9)	0.676
Previous stroke	40 (21.5)	8 (25.0)	32 (20.8)	0.597
TIA	3 (1.6)	0	3 (1.9)	0.426
Smoking	4 (2.2)	2 (6.3)	2 (1.3)	0.079
*Stroke Type*				
Ischemic	138 (84.4)	27 (72.5)	111 (74.6)	0.559
Hemorrhagic	35 (19)	4 (12.5)	31 (20.3)

CAD - coronary artery disease, DM - diabetes mellitus, HTN – hypertension

**Table 2:** Locations of hemorrhage in cases of hemorrhagic stroke. N=35

Locations	n	(%)
Lobar	13	(37.1)
Baseline	18	(51.4)
***Gangliothalamic***		
Infratentorial	2	(5.7)
Intraventricular extension	10	(28.6)

These locations are not mutually exclusive which explains why the total number does they add up to 35

**Table 3** shows some parameters related to patient management and stroke outcome. The median time from symptoms onset to presentation was 7.4 hours. All patients had CT scan imaging while 35 (18.8%) had MR imaging in addition. Only five patients received intravenous tissue plasminogen activator (IV tPA); all were treated within the KAMC Hajj Stroke Hotline program. The most common reasons for not giving tPA were delay in arrival to the hospital (72.2%) and the presence of tPA contraindications (19%).

**Table 3:** Stroke management parameters and patient outcome.

Parameters	All cases n=186	Saudis n=32	Non-Saudis n=154	*P*-value
	Median (IQR)			
MRI performed n (%)	35 (18.8)	10 (31.3)	25 (16.4)	0.052
Onset to arrival time in hours	n=64 7.4 (3.0-24.0)	n=14 3.0 (1.8-9.1)	n=50 9.0 (4.1-24.0)	0.026*
Time from arrival to imaging in hours	n=175 1.5 (0.4-13.9)	n=29 0.8 (0.2-3.3)	n=146 1.6 (0.5-17.9)	0.062
Hospital stay (days)	n=185 4.0 (2.0-6.5)	n=32 3.0 (2.0-6.8)	n=153 4.0 (2.0-6.5)	0.414
Death n (%)	21 (11.3)	3 (9.4)	18 (11.7)	0.707

* Statistically significant at *p*<0.05, n above individual variables refers to the number of cases with available data about that variable

**Table 4** summarizes the reasons for not giving tPA to ischemic stroke patients. Among reasons listed as “Others” is the absence of tPA in the hospital. The median length of hospital stay was 4 days. Twenty-one patients (11.1%) died during hospitalization. **Table 5** summarizes patients’ characteristics according to mortality status. The association of various risk factors with mortality was examined in a logistic regression model. The only factor that approached statistical significance in predicting mortality was the type of stroke with an odds ratio of 1.62 of hemorrhagic versus ischemic stroke (95% CI: 0.97-2.70; *p*=0.063). The latter association was explored excluding cases with non-specified stroke type. Other factors like age, gender and nationality (Saudi versus other) did not show statistically significant association with mortality.

**Table 4:** Reasons for not giving tPA. N=138

Reasons	n	(%)
Delay of patient arrival to hospital	99	(71.7)
Delay inside the hospital	5	(3.6)
Contraindication	5	(3.6)
Other reason	3	(2.2)
Reason not listed	26	(18.8)

**Table 5:** The characteristics of registered stroke cases according to mortality status.

Parameters	Survived n=165	Died n=21	*P*-value
	n (%)	
Age Mean±SD	60.3±12.9	64.3±12.9	0.184
Males n (%)	108 (65.5)	11 (52.4)	0.240
Saudis	29 (17.6)	3 (14.3)	0.707
BMI Mean±SD	29.3±28.5	25.8±4.1	0.714
CAD	22 (13.3)	1 (4.8)	0.440
DM	73 (44.2)	3 (14.3)	0.009*
HTN	98 (59.4)	8 (38.1)	0.063
AFIB	4 (2.4)	1 (4.8)	1.0
Hyperlipidemia	4 (2.4)	0	1.0
Previous stroke	35 (21.2)	5 (23.8)	1.0
TIA	3 (1.8)	0	1.0
Smoking	4 (2.4)	0	1.0
Ischemic	126 (76.8)	12 (57.1)	
Hemorrhagic	28 (17.1)	7 (33.3)	0.240
Non-specified	10 (6.1)	2 (9.5)	

CAD - coronary artery disease, DM - diabetes mellitus, HTN - hypertension, SD - standard deviation,

* statistically significant at *p*<0.05

## Discussion

In this registry, we sought to explore the incidence of stroke among the pilgrims and predict the factors associated with presentation and management during Hajj. We found that pilgrims had a relatively high incidence rate of stroke compared to the expected figures in the region.^5^ However, the incidence reported in the current study was calculated using as a denominator the official number of pilgrims reported by the Saudi authorities. The incidence may have been thus overestimated because the actual number of pilgrims is probably higher. On the other hand, fatal stroke victims and missed stroke cases were not included in the numerator leading our estimate to be an underestimate of the true incidence. The effects of these 2 counteracting factors may result in our calculated incidence to be a good estimation of the true incidence.

There is a paucity of research on stroke in Saudi Arabia generally, and among pilgrims specifically.^6^ We found only one study examining the care of stroke patients during Hajj.^3^ That study was limited to the characteristics of pilgrims from a single country and thus provide no insight concerning stroke incidence during Hajj. This registry included all pilgrims admitted with stroke at Makkah to provide an accurate estimate of the true incidence of stroke during Hajj. Another limitation of the study by Azarpazhooh et al^3^ is that they did not examine relationships among patients’ characteristics, diagnostics testing, treatments and outcomes. We addressed this issue by collecting high quality data for all acute stroke patients, and by investigating the relations of many factors (e.g., patients’ characteristics) to the stroke care management during Hajj.

We found that the peak incidence of the strokes was on the tenth day of Hajj. One possible explanation for this finding is that physical stress, resulted from performing the Hajj rituals on the tenth day of Hajj, could have caused a sudden rise in blood pressure and heart rate. This eventually might have led to atrial fibrillation, which is a leading cause for stroke. Atrial fibrillation can be paroxysmal in nature which explains why we did not detect AF in many of our patients. Another potential explanation is that pilgrims were busy performing the rituals on the tenth of Hajj, and pilgrims with chronic diseases (e.g., diabetes and hypertension) might have not been compliant to their medications, and this could have led to the increase of stroke incidence on the mentioned day.^7^

The majority of the stroke patients were admitted to two hospitals: King Abdulaziz hospitals and Al Noor specialist hospital. King Abdullah Medical City in Makkah introduced the hotline program for ischemic stroke during 1436 Hajj. Thus, recruiting a sufficient number of strokes specialists in these hospitals during Hajj time is needed. Rapid coordination for transferring stroke patients by medical air transport among hospitals is important in order to treat patients with thrombolytic therapies which will have significant impact on the outcome of stroke care during Hajj.^8^

Thrombolytic therapy in the form of IV tPA was given to 5 patients only; all in KAMC. Although treatment with IV tPA was given to a small number of patients during Hajj, this number is suspected to be a significant increase from previous years. The main reason for not giving IV tPA was the late arrival to the hospital. This is likely attributed to the lack of awareness for stroke signs, symptoms, risk factors and treatment. This lack of awareness of stroke symptoms may also explain the observed delays in arrival to hospital between the Saudis and non-Saudi pilgrims. Our results confirm the need for a more extensive public education for pilgrims to increase awareness of stroke symptoms and risks and the presence of treatment modalities that can help alleviate stroke symptoms only if given in time.^9^

The hospital mortality related to stroke in our sample was comparable to available literature.^10^ Univariate logistic regression showed an increased risk of mortality with hemorrhagic versus ischemic stroke yet the association failed to reach statistical significance. Other factors like age and gender did not show a significant association with mortality. Cross tabulation shows a significantly smaller percentage of diabetics and numerically smaller percentage of hypertensives among those who died. This may be due to a confounding effect of the type of stroke, yet multivariate regression could not be attempted with the current set of data because of the relatively small number of cases. This calls for more data collection in the subsequent years and also calls for launching of a National Stroke Registry, which will permit in depth analysis of the risk factors in the Hajj setting and in the Saudi population.

In conclusion, we present the findings of the first multi-center Hajj stroke registry which was centered and organized by King Abdullah Medical City in Makkah. Our findings identify the groups at high stroke risk and the peak incidence time so that organizational and educational interventions can be planned in future years to improve the care of this patients’ population.
